# Timeseries of multispectral and radar data and vegetation indices from Sentinel-1, Sentinel-2 and Landsat-8 at field scale

**DOI:** 10.1016/j.dib.2026.112902

**Published:** 2026-06-06

**Authors:** Frédéric Frappart, Bertrand Ygorra, Frédéric Baup, Alexis Martin-Comte, Kevin Gross, Rémy Fieuzal, Clément Battista, Serge Riazanoff

**Affiliations:** a ISPA, UMR1391 INRAE/Bordeaux Science Agro, 33140 Villenave d’Ornon, France; b Institut National de Recherche en Sciences et Technologies du Numérique, 33405 Talence, France; c Centre d’Etudes Spatiales de la Biosphère, University of Toulouse, CNES/CNRS/IRD/INRAE, Toulouse, France; d VisioTerra, 77420 Champs-Sur-Marne, France

**Keywords:** Time series, SAR backscatter, NDVI, Agricultural fields, Sentinel-1, Sentinel-2, Landsat-8

## Abstract

Monitoring agricultural lands is crucial for achieving food security. Earth Observation (EO) has recently become an essential tool to reach this goal owing to advances in spatial and temporal resolutions as well as radiometric accuracy of the current satellite sensors. Yet, the size of the datasets and the need to preprocess them limit their dissemination to some scientific communities and in direction to stakeholders. The dataset presented here is composed of pluriannual time series of variables acquired by high spatial (10-30 m) and medium to high temporal resolutions (a few days combining images from different swaths) from radar (Sentinel-1) and multispectral (Landsat-8 and Sentinel-2) EO satellites along with commonly used vegetation indices and biophyscial variables averaged over more than 1,400 agricultural fields. Data were made available in the framework of a project aimed at developing the application of numeric tools on EO data for agroecology purposes. This dataset was made publicly available owing to its easyness to use and interest for agronomists, environmentalists as well as economics and politics stakeholders.

Specifications Table

**Table:** 

Subject	Agricultural Sciences ; Agronomy and Crop Science
Specific subject area	Phenology and growth remotely sensed variables of numerous crop types at field level
Type of data	Table (in .csv format)
Data collection	Sentinel-1 (L1 GRD in IW mode) and 2 (L2A) images were downloaded from CopernicusSciHub, Landsat-8 (L2SP) images from USGS. Information on agricultural field locations and crop types come from the French database Registre Parcellaire Graphique. The merging of the two sources of information was performed by VisioTerra.
Data source location	Fields contained in two circles of 6 km of radius and centered around the Lamasquère (43.4992°N, 1.2358°E) and Auradé (43.5495°N, 1.1061°E) ICOS stations in Occitanie region, southwest of France
Data accessibility	Repository name: Recherche.data.gouv.fr Data identification number: 10.57745/QLKA8K Direct URL to data: 10.57745/QLKA8K
Related research article	Baup F., Fieuzal, R., Ygorra, B., Frappart, F., Riazanoff, S., Martin-Comte, A., & Gorrab, A. (2026). 6 Years of SAR (Sentinel-1) and Optical (Sentinel 2, Landsat-8) Acquisitions over Agricultural Surfaces in Southwestern France. Remote Sensing, 18(5), 790, 10.3390/rs18050790.

## 1 Value of the Data

• This dataset provides multi-year temporal evolution of remote sensing observations and derived vegetation indices from Sentinel-1 (radar), Sentinel-2 and Landsat-8 (multispectral) spatially averaged at field scale ca. 1,400 fields located in the southwest of France and associated to crop type.
• It allows investigation on spatio-temporal changes on crop phenology and growth stages for the same crop type or between crop types.
• Combined with climate and environmental information, this dataset can be used to analyze the impact of climate and anthropogenic changes on agriculture.
• This dataset can be used for calibating and validating agrometorological models on a wide range of crops.
• This resource constitutes a valuable an easy-to-handle database for non remote sensing experts.

## 2 Background

Monitoring agricultural areas requires obtaining information on the surface dynamics, including soil and vegetation properties. Different monitoring methods can be used, from ground measurements to remotely sensed data, including climate or crop modeling [1].

The dataset presented here gives an easy access to information from high resolution multispectral and Synthetic Aperture Radar (SAR) images at field-scale. It results from the average of:

• either the surface reflectances from Sentinel-2 or Landsat-8 or 9 [2] and some spectral indices including Normalized Difference Vegetation Index (NDVI) [3],
• or the backscatter at C-band Vertical Horizontal (VH) and Vertical Vertical (VV) polarizations from Sentinel-1 [4], their ratio, the radar vegetation index (RVI) [5] on the area of every field inventoried in an administrative information layer in raster format containing the agricultural field identifier and the crop type.

The dataset made available consists in a series of files for each field contained in the study area located in the southwest of France containing the timeseries of either SAR (Sentinel-1) or multispectral (Landsat-8, Sentinel-2) data, separately provided between January 2016 and December 2021.

## 3 Data Description

The dataset made available on the Recherche.data.gouv.fr website (https://entrepot.recherche.data.gouv.fr/) is composed of a series of .csv format files named using the following pattern : Satellite identifier and field identification number separated by « _ ». Satellite identifier is either s1, s2 or l8 standing for Sentinel-1, Sentinel-2, Landsat-8, respectively. It is available over all fields located in two circles of 6 km of radius and centered around the Lamasquère and Auradé stations in Occitanie region, southwest of France. The field identification comes from the Registre Parcellaire Graphique (RPG), a Geographical Information System (GIS) allowing the identification of the agricultural plots, providing the field delineation and the crop types, and the French administration Agence de Services et de Paiement (ASP) [6].

The files containing the time series have the following structure :

• information about the agricultural plot and similar for all the satellite missions;
• specific information related to each satellite mission.

They are formatted with a header line and columns containing the datasets as follows :

### 3.1 Common information

The common information to any file is contained in the 5 first columns :

• DATE : date of the image acquisition in YYYYMMDD format;
• AREA_HA : float giving the field area (ha) estimated from the vector representing each field;
• CODE_CULTU : character string giving the crop type designation in the RPG;
• LIBELLE : character string giving the crop type in French;
• LIBELLE_EN : character string giving the crop type in English.

### 3.2 Satellite mission specific information

Sentinel-1

**Fig. 1:**
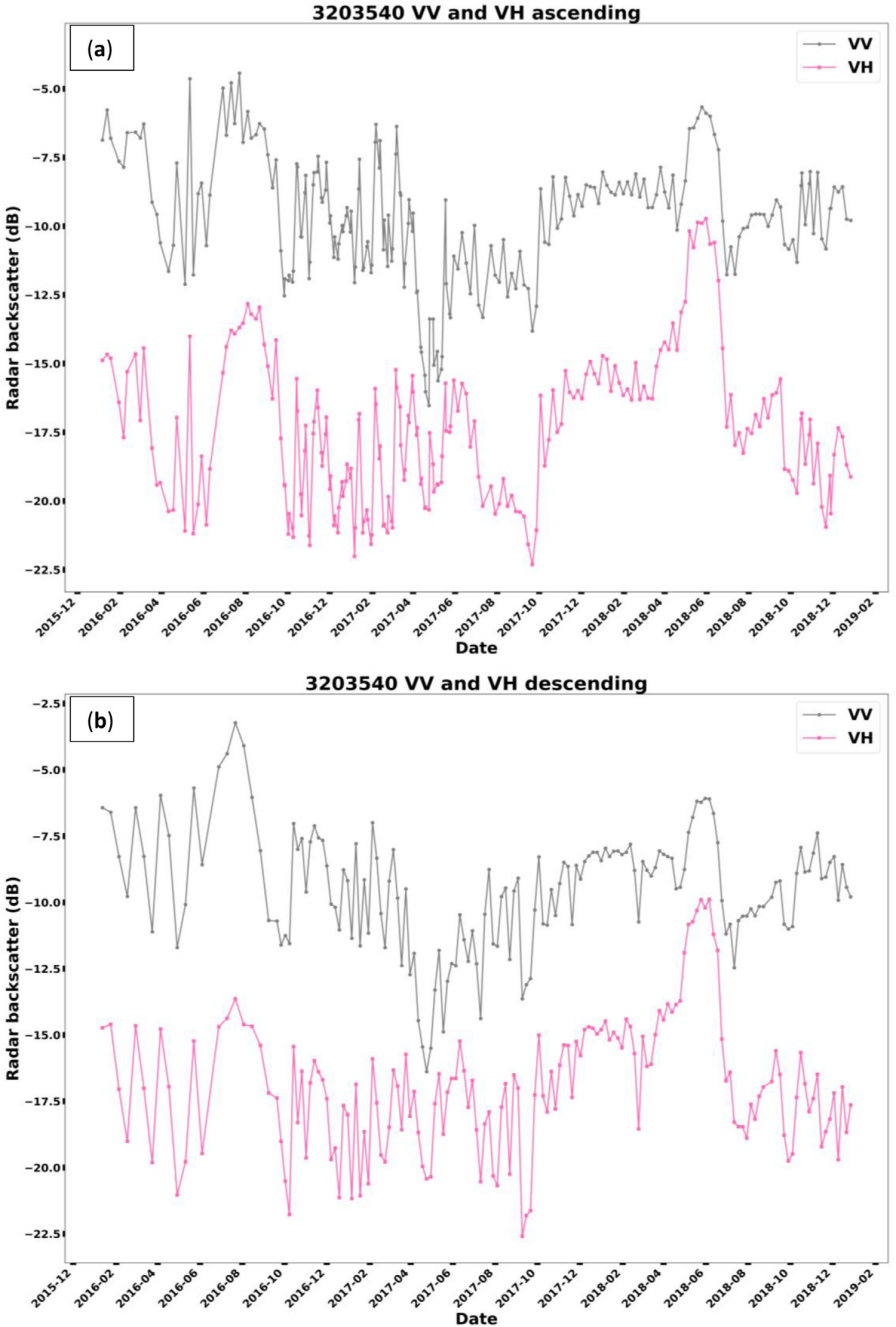
Time series of Sentinel-1 backscatter from 2016 to 2018 at VH (black) and VV (pink) polarizations over RPG field number 3203540 for: (**a**) ascending and (**b**) descending orbits.

The information specific to Sentinel-1 files is contained in four columns :

• ORBIT NUMBER : integer giving the orbit number;
• ORBIT TYPE : character A and D for ascending and descending orbits, respectively ;
• VV: float giving the C-band backscatter (dB) at VV polarization;
• VH : float giving the C-band backscatter (dB) at VH polarization.
• ratio : float giving the ratio of C-band backscatter at VV and VH polarization (VV/VH) ;
• RVI : float between - 1.0 and 1.0 giving the Radar Vegetation Index (RVI value ;

An example of time-series of Sentinel-1 SAR C-band backscatter at VH and VV polarizations over field RPG number 3203540 is presented in Fig. 1. Using the information contained in the file, it is possible to separately analyze the signal from Sentinel-1 ascending and descending orbits over the considered field using the information contained in column « ORBIT TYPE ».

Sentinel-2

The information specific to Sentinel-2 files is contained in twenty columns :

• NDVI : float between - 1.0 and 1.0 giving the NDVI value;
• FCOVER : float between - 1.0 and 1.0 giving the fraction of vegetation cover (fCover);
• LAI : float giving the leaf area index (LAI) in m^2^.m^−2^ ;
• FAPAR : float giving the fraction of Absorbed Photosynthetically Active Radiation (fAPAR);
• NDWI-SM : float between - 1.0 and 1.0 giving the Normalized Difference Water Index – SM (NDWI-SM) value;
• VIEWING AZIMUTH ANGLE : float between 0.0 and 360.0 giving the azimuth angle (°) in which the field is seen;
• VIEWING ZENITH ANGLE : flot between 0.0 and 90.0 giving the zenith angle (°) in which the field is seen;
• SUN AZIMUTH ANGLE : float between 0.0 and 180.0 giving the sun azimuth angle (°) for the considered field;
• SUN ZENITH ANGLE : float between 0.0 and 90.0 giving the sun zenith angle (°) for the considered field;
• B02_BLEU_490 : float between 0.0 and 1.0 giving the reflectance in the blue band at 492 nm;
• B03_VERT_560 : float between 0.0 and 1.0 giving the reflectance in the green band at 560 nm;
• B04_ROUGE_665 : float between 0.0 and 1.0 giving the reflectance in the red band at 665 nm;
• B05_VRE1_705 : float between 0.0 and 1.0 giving the reflectance in the vegetation red edge band at 704 nm;
• B06_VRE2_740 : float between 0.0 and 1.0 giving the reflectance in the vegetation red edge band at 740 nm;
• B07_VRE3_783 : float between 0.0 and 1.0 giving the reflectance in the vegetation red edge band at 780 nm;
• B08_NIR1_842 : float between 0.0 and 1.0 giving the reflectance in the near infrared (NIR) band at 833 nm;
• B8A_NIR2_865 : float between 0.0 and 1.0 giving the reflectance in the narrow NIR band at 864 nm;
• B11_SWIR1_1610 : float between 0.0 and 1.0 giving the reflectance in the short wave infrared (SWIR) band at 1610 nm;
• B12_SWIR2_2190 : float between 0.0 and 1.0 giving the reflectance in the SWIR band at 2200 nm;
• CLOUD_COVER_% : integer between 0 and 100 giving the percentage of cloud cover.

**Fig. 2:**
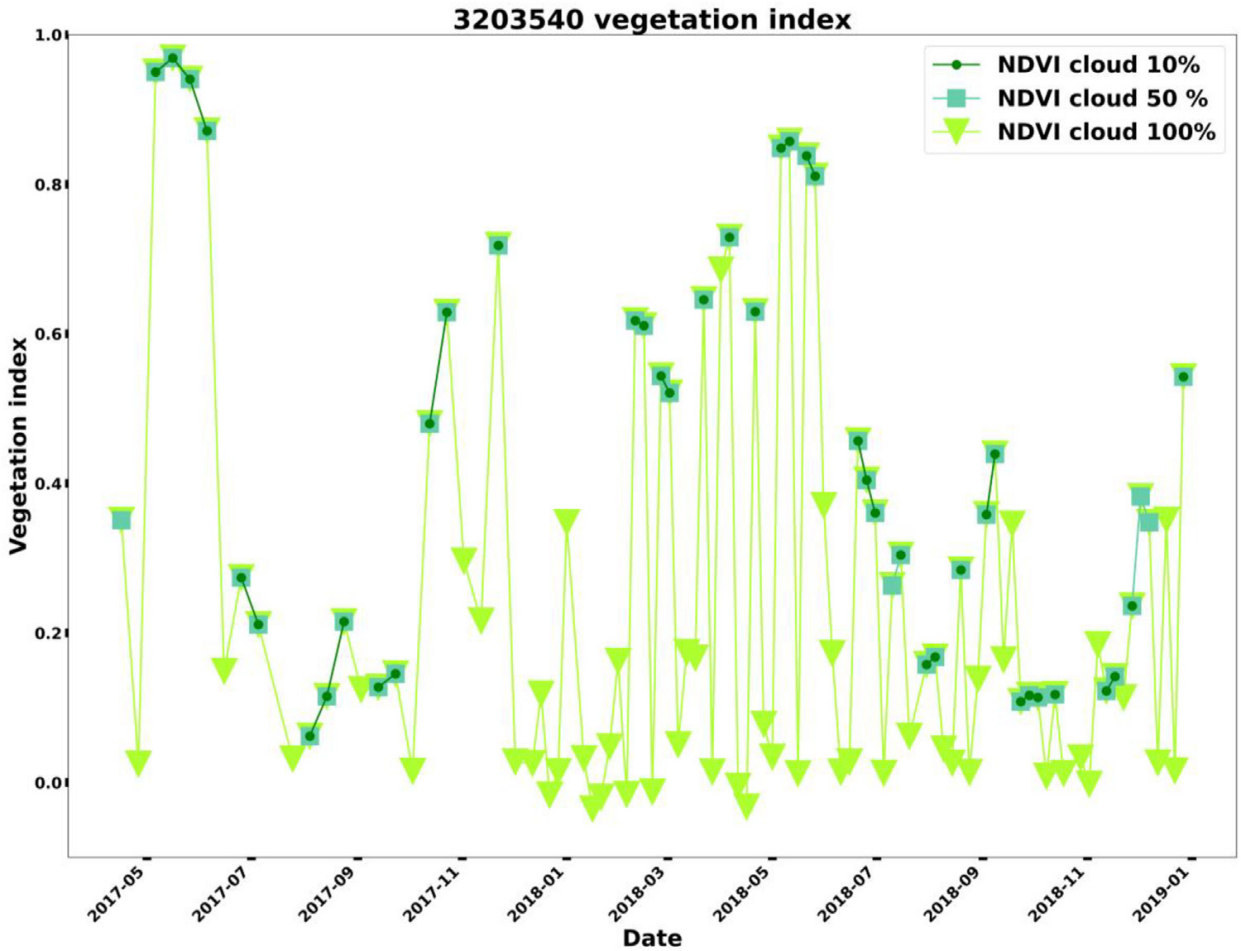
Time series of Sentinel-2 NDVI for 10% (dark green dots), 50% (green squares) and 100% (light green triangles) cloud cover from 2017 to 2018 over RPG field number 3203540.

An example of time-series of NDVI derived from Sentinel-2 acquisitions for different cloud cover percentages (10, 50, and 100%) over field RPG number 3203540 is presented in Fig. 2. Using the information contained in the file, it is possible to select the cloud cover the image containing the considered field using the information from column « CLOUD_COVER_% ». Depending on the selected threshold, the number of data changes.

Landsat-8

The information specific to Landsat-8 files is contained in twenty columns :

• NDVI : float between - 1.0 and 1.0 giving the NDVI value;
• NDWI-SM : float between - 1.0 and 1.0 giving the NDWI-SM value;
• VIEWING AZIMUTH ANGLE : float between 0.0 and 360.0 giving the azimuth angle (°) in which the field is seen;
• VIEWING ZENITH ANGLE : float between 0.0 and 90.0 giving the zenith angle (°) in which the field is seen;
• SUN AZIMUTH ANGLE : float between 0.0 and 180.0 giving the sun azimuth angle (°) for the considered field;
• SUN ZENITH ANGLE : float between 0.0 and 90.0 giving the sun zenith angle (°) for the considered field;
• B02_BLEU_480 : float between 0.0 and 1.0 giving the reflectance in the blue band at 492 nm;
• B03_VERT_560 : float between 0.0 and 1.0 giving the reflectance in the green band at 560 nm;
• B04_ROUGE_665 : float between 0.0 and 1.0 giving the reflectance in the red band at 665 nm;
• B05_NIR_842 : float between 0.0 and 1.0 giving the reflectance in the NIR band at 833 nm;
• B11_SWIR1_1610 : float between 0.0 and 1.0 giving the reflectance in the short wave infrared (SWIR) band at 1610 nm;
• B05_SWIR2_2190 : float between 0.0 and 1.0 giving the reflectance in the SWIR band at 2200 nm;
• CLOUD_COVER_% : integer between 0 and 100 giving the percentage of cloud cover.

**Fig. 3:**
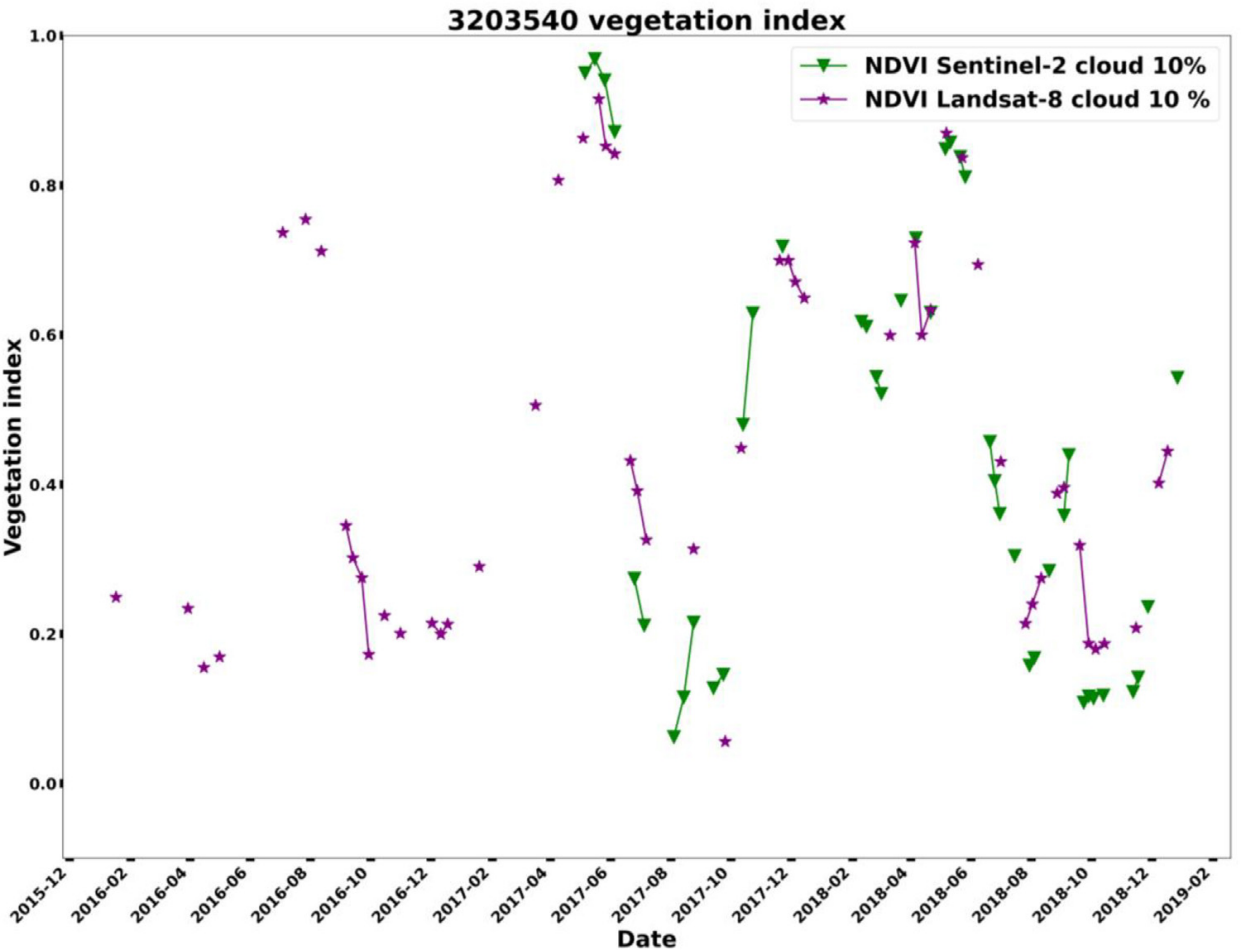
Time series of Sentinel-2 (dark green triangles) and Sentinel-8 (purple stars) NDVI for 10% cloud cover from 2016 to 2018 over RPG field number 3203540.

An example of time-series of NDVI derived from Sentinel-2 and Landsat-8 acquisitions for a cloud cover of 10% over field RPG number 3203540 is presented in Fig. 3.

## 4 Experimental Design, Materials and Methods

### 4.1 Site description

**Fig. 4:**
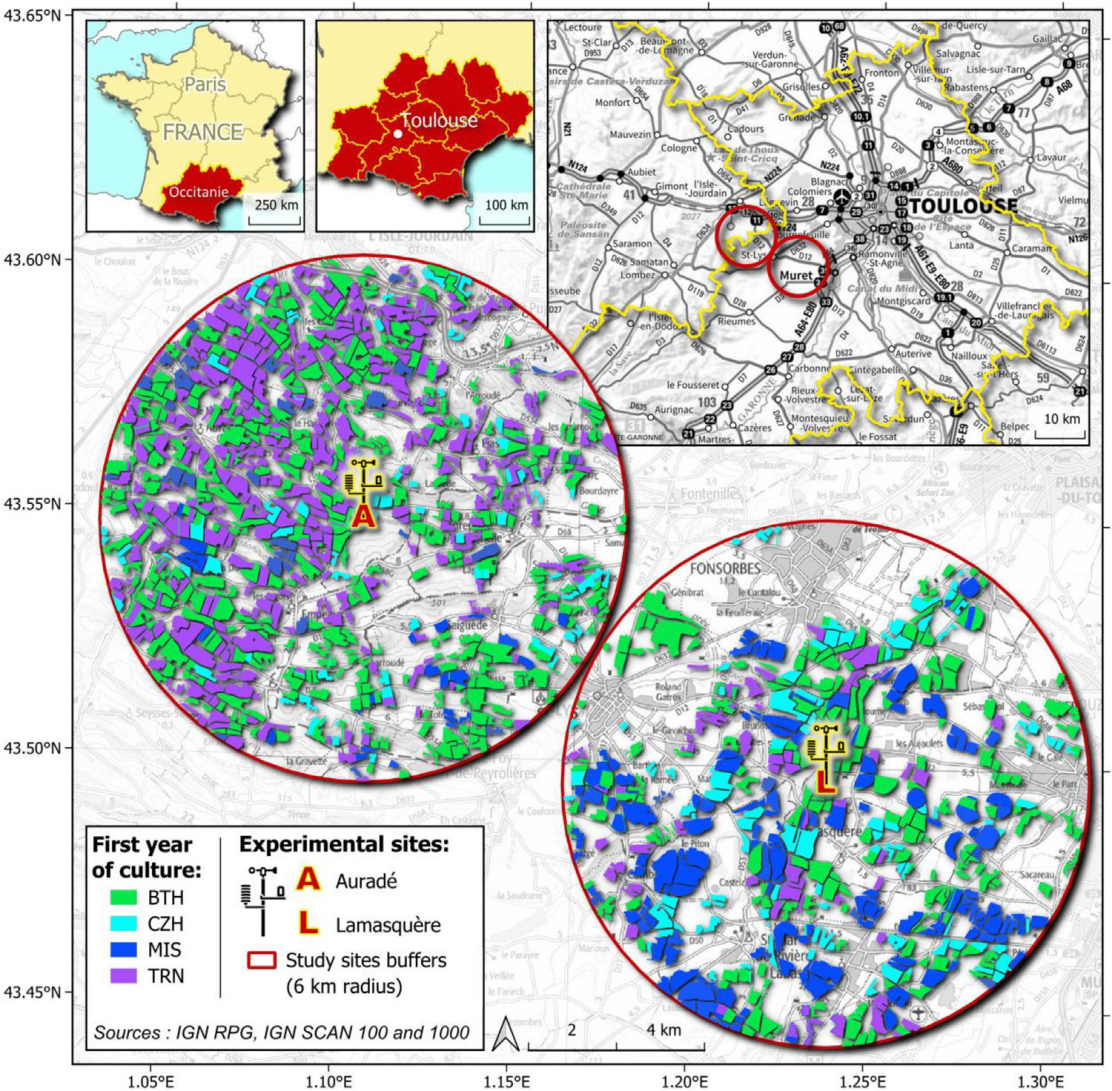
Location of the study area centered around Auradé and Lamasquère ICOS stations, Occitanie region (France).

The study area is composed of 1,400 fields located in two circles of 6 km of radius and centered around the Lamasquère and Auradé stations in Occitanie region, southwest of France (Fig. 4). These monitoring stations were chosen as they are part of numerous national and international observation networks such as Regional Observatory South-West (RSO SW - https://osr.cesbio.cnrs.fr/), Integrated Carbon Observation System (ICOS - https://icos-france.fr/), and Joint Experiment for Crop Assessment (JECAM - https://jecam.org/), and their data were used for comparison with EO data in a large number of studies (e.g., [[7], [8], [9], [10]]).

**Fig. 5:**
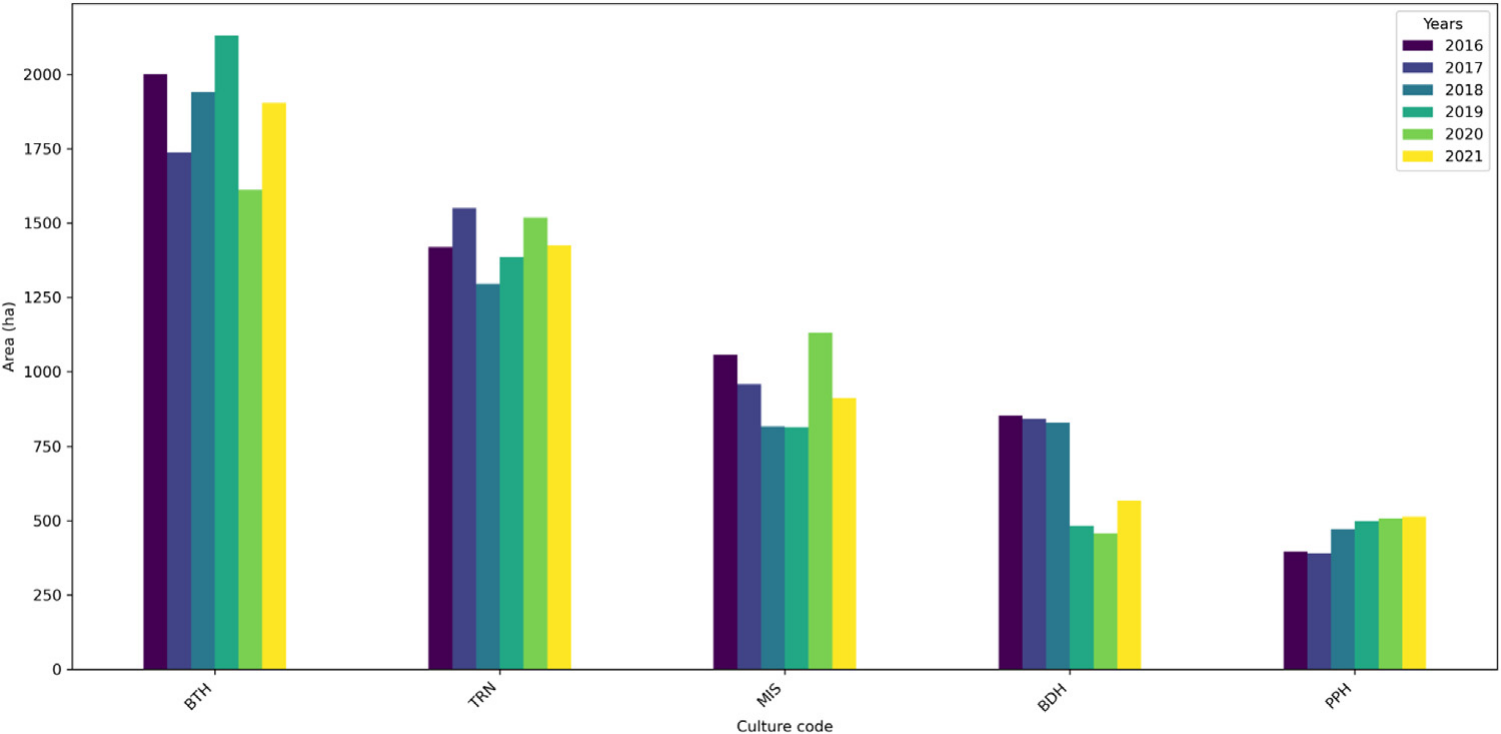
Area (ha) occupied by the five major crop types present in the study area (soft winter wheat – BTH, sunflower – TRN, corn – MIS, hard winter wheat - BDH, permanent pasture – predominantly grass) over 2016-2021.

These fields are mostly covered with soft winter wheat, sunflower, corn, hard winter wheat, and permanent pastures and the study area is subject to important crop rotation [[11], [12], [13]], as illustrated in Fig. 5 with the annual area of the five major crop types over the study area between 2016 and 2021.

### 4.2 Material and methods

The method consists in merging information from raster datasets (satellite images from Sentinel-1, Sentinel-2, and Landsat-8) with high spatial (between 10 and 30 m) medium to high temporal (high or equal to 13 days) resolutions with vector information annually updated to account for changes in plot delineation and above all in crop types as the study area is subject to important crop rotation [[11], [12], [13]].

For each of the agricultural fields of the study area, information about the field (area and crop type among the 61 available – see for the nomenclature) is extracted from the RPG database for the year corresponding to the date of acquisition of every image from any satellite mission. These information are merged with the generic information related to the satellite mission considered (e.g., date of image acquisition, ascending or descending orbits, ...), the average of the EO variables presented in subsection *3.2. Satellite specific information* in the boundary of the field. Note that not all the fields were not cultivated during the study period due to the important rotation. When the fields were not cultivated, as no information was present in the RPG, no information was provided in the file. For this reason, the length of the files is different and do not necessarily start or end at the same date. This operation repeated for :

• all the files over the study period (here 2016-2021) to generate a time series per satellite mission (i.e., Sentinel-1, Sentinel-2, Landsat-8);
• the three satellite missions considered here;
• the agricultural fields of the study area;

was performed by VisioTerra. The complete database made available is composed of a total of 4,212 files, with 1,400 different fields.

The following EO datasets were used :

• Sentinel-1 C-band (5.045 GHz) images at VH and VV polarizations acquired in Interferometric Wide-swath (IW) mode with a ground resolution of 5×20 m and a temporal in Ground Range and Multi-Look Detected (GRD) format and a temporal resolution of 6 days over Europe [4]. The processing of Sentinel-1 images was performed through VtWeb (https://visioterra.org/VtWeb/). It includes orbit correction followed by Thermal Noise Removal [14] and terrain flattening as terrain correction to reduce topological effects and to remove incidence angle effect [15], using the 5-meters resolution Digital Elevation Model (DEM) provided by the French geographic institute (IGN), as described in [16] to obtain terrain corrected backscatter (γ^0^) at 10 m spatial resolution. They were downloaded from VtWeb. RVI was derived from terrain corrected backscatter using the following equation [5]:
  (1) $$RVI\;=\;\frac{4\gamma_{VH}^{0}}{\gamma_{VV}^{0}+\gamma_{VH}^{0}}$$
  where $\gamma_{VH}^{0}$ and $\gamma_{VV}^{0}$ are the terrain corrected backscatter at VH and VV polarizations, respectively.
• Sentinel-2 images acquired by the MultiSpectral Imager (MSI) at spatial resolutions of 10 m (for B02_BLUE, B03_GREEN, B03_RED, B08_NIR1), and 20 m (B06_VRE1, B07_VRE2, B08_VRE3, B8A_NIR2, B11_SWIR1, B12_SWIR2) and a temporal resolution of 6 days over Europe [17]. Two levels of products were used : Level 1C (L1C) Top of the Atmosphere reflectance and Level 2A (L2A) Bottom of the Atmosphere (BOA) reflectance processed from L1C using Sen2Cor [18]. They were downloaded from VtWeb (https://visioterra.org/VtWeb/). Surface Reflectances at 10 m and 20 m spatial resolution from L2A were used. NDVI [3] and NDWI SM [19] were derived using the following equations :
  (2) $$\text{NDVI}=\frac{\rho_{Red}\;-\;\rho_{NIR}}{\rho_{Red}\;+\;\rho_{NIR}}$$
  (3) $$\text{NDWI}=\frac{\rho_{Green}\;-\;\rho_{NIR}}{\rho_{Green}\;+\;\rho_{NIR}}$$
  where ρ_Green_, ρ_Red_, and ρ_NIR_ are the reflectances in the green, red, and NIR, respectively.
  Sentinel-2 LAI, fAPAR, and fCOVER biophysical variables were generated from L1C TOA images using the L2B processor [20].
• Landsat-8 images acquired by the Operational Land Imager (OLI) at spatial resolution of 30 m and temporal resolution of 16 days for Band 1 Coastal aerosol, Band 2 Blue, Band 3 Green, Band 4 Red, Band 5 NIR, Band 6 SWIR1, Band 7 SWIR 2 and Band 9 Cirrus [2]. Surface reflectances from Level-2 Science Products (L2SP) were downloaded from VtWeb (https://visioterra.org/VtWeb/). NDVI and NDWI were derived using (2) and (3).

## Limitations

Not applicable

## Ethics Statement

The authors have read the ethical requirements for publication in Data in Brief and confirm that the current work does not involve human subjects, animal experiments, or any data collected from social media platforms.

## CRediT Author Statement

**Frédéric Frappart :** Conceptualization, Methodology, Original draft preparation, Writing - Reviewing and Editing. **Bertrand Ygorra :** Conceptualization, Methodology, Data curation, Original draft preparation, Writing - Review and Editing. **Frédéric Baup :** Conceptualization, Methodology, Writing - Reviewing and Editing. **Alexis Martin-Comte :** Data curation, Writing - Reviewing and Editing. **Kévin Gross :** Data curation, Writing - Reviewing and Editing. **Rémy Fieuzal :** Conceptualization, Methodology, Writing - Reviewing and Editing. **Clément Battista :** Data curation, Writing - Reviewing and Editing. **Serge Riazanoff :** Conceptualization, Methodology, Writing - Review and Editing.

## Data Availability

Recherche.data.gouv.frTimeseries of multispectral and radar observations and vegetation indices from Sentinel-1, Sentinel-2 and Landsat-8 at field scale (Original data).
